# The impact of sentinel lymph node biopsy on the quality of life in patients with oral cavity squamous cell carcinoma

**DOI:** 10.1016/j.bjorl.2020.11.015

**Published:** 2020-12-27

**Authors:** Marco Roberto Seferin, Fábio Roberto Pinto, Ana Kober Nogueira Leite, Rogerio Aparecido Dedivitis, Marco Aurélio Vamondes Kulcsar, Claudio Roberto Cernea, Leandro Luongo de Matos

**Affiliations:** Universidade de São Paulo, Faculdade de Medicina, Departamento de Cirurgia de Cabeça e Pescoço, São Paulo, SP, Brazil

**Keywords:** Sentinel lymph node biopsy, Squamous cell carcinoma, Neck dissection, Mouth neoplasms, Quality of life

## Abstract

**Introduction:**

Sentinel lymph node biopsy is a proven method for staging the neck in patients with early oral cavity squamous cell carcinoma because it results in less comorbidity than the traditional method of selective neck dissection, with the same oncological results. However, the real effect of that method on the quality of life of such patients remains unknown.

**Objective:**

The present study aimed to evaluate the quality of life of patients with oral cavity squamous cell carcinoma T1/T2N0 submitted to sentinel lymph node biopsy compared to those that received selective neck dissection.

**Methods:**

Cross-sectional study including 24 patients, after a 36 month follow-up, 15 of them submitted to the sentinel lymph node biopsy and 9 to selective neck dissection. All patients answered the University of Washington quality of life questionnaire.

**Results:**

The evaluation of the questionnaires showed a late worsening of the domains appearance (*p* = 0.035) and chewing (*p* = 0.041), as well as a decrease of about 10% of general quality of life (*p* = 0.025) in patients undergoing selective neck dissection ​​in comparison to those undergoing sentinel lymph node biopsy.

**Conclusion:**

Patients with early-stage oral cavity squamous cell carcinoma undergoing sentinel lymph node biopsy presented better late results of general quality of life, mainly regarding appearance and chewing, when compared to patients submitted to selective neck dissection.

## Introduction

The presence of cervical metastases is considered to be the main factor for poor prognosis in patients with squamous cell carcinomas (SCC) of the head and neck.^1^, ^2^ Regarding the oral cavity, primary tumors in the early stage (T1, T2), without clinical evidence of neck disease (cN0), may present an incidence of occult metastases ranging from 10% to 50%.^3^, ^4^ Therefore, neck management plays a key role in therapeutic planning.

In previous decades, for patients with T1/T2 N0 oral squamous cell carcinoma, the discussion about neck management was polarized between selective neck dissection (SND) and surveillance with strict observation of the patient's evolution (“watchful waiting”), reserving neck dissection only for those patients who would develop metastatic lymph node disease during followup.^5^

Those who favor selective neck dissection base their arguments on apparently better oncological outcomes.^6^, ^7^ Undiagnosed lymph node disease increased the risk of recurrence and decreased survival at 5 years from 82% to 53%.^8^ In addition, in a prospective, randomized study, D’Cruz et al.^9^ showed that SND for patients with early stage oral cavity SCC had a higher disease-free survival from 45.9% to 69.5% when compared to the watchful waiting method.

On the other hand, the defenders of surveillance draw attention to possible sequelae due to the neck dissection.^10^, ^11^ SND may contribute to anesthesia or paresthesia of the ear and neck, lower lip paralysis, neck pain, shoulder disability, lymphedema, neck retraction and changes in the individual's appearance.^12^ Leipzig et al.^13^ reported that 30% of patients who underwent SND developed postoperative shoulder syndrome, even when the accessory nerve was preserved. In addition, approximately 70% of patients did not have evidence of lymph node involvement after histopathological examination of the SND product.^14^

Based on this, the sentinel lymph node biopsy (SLB) appears to be an alternative option that is oncologically safe with less morbidity than SND.^6^, ^15^ Most clinically N0 patients can be spared from neck dissection, which is only used for the regional staging of pN0 patients. Thus, the SLB brings the perspective of having a lower impact on the quality of life in this group of patients, without compromising oncological radicality. However, the real effect of SLB on the quality of life for these patients remains unknown.

Therefore, the aim of the present study was to evaluate the quality of life of patients with T1/T2 N0 oral cavity squamous cell carcinoma receiving SLB, compared to those with the same clinical stage who underwent SND.

## Methods

A cross-sectional study was performed that included patients with T1/T2 N0 squamous cell carcinoma of the oral cavity who underwent a sentinel lymph node biopsy or cervical neck dissection levels I, II, III. After a 36 month follow-up, 24 patients were enrolled, 15 of them received SLB and 9 had SND.

The study was approved by the IRB at number 018/15 and all patients signed the informed consent form. All patients answered the University of Washington quality of life questionnaire, 4th edition, translated into Portuguese and validated for use in the Brazilian territory.^16^ It is considered the most internationally used instrument for evaluation of the quality of life of patients during the treatment of cancer of the mouth, larynx and pharynx.^17^

Patients who had relapsed disease after the first treatment or evidence of a second primary tumor were excluded from the study. In addition, patients who required a composite approach with the neck region for resection of the primary tumor, even in T1/T2 N0 clinical staging, or those initially submitted to SLB but required neck dissection a second time were also not eligible for the study.

Patients operated upon in the years 2014 and 2015, by both techniques, were invited to complete the quality of life questionnaire 36 months after surgery. In these two years, 51 patients underwent SLB or SND, but 15 and 9 individuals were interviewed respectively, totaling 24 patients who were included in the late quality of life assessment. Of the remaining 27, eleven patients died, seven had undergone radical neck dissection after SLB, five presented as relapsing at the time of the interview or had undergone a new surgical procedure in the head and neck region, and only four were not located.

Clinical characteristics such as gender, age, primary tumor subsite, histological type, pathological staging (pTNM – AJCC seventh edition), presence of positive margin, tumor thickness, presence of perineural and/or angiolymphatic infiltration and adjuvant treatment were collected. The performance status evaluation at the time of application of the questionnaire was performed by the ECOG (Eastern Cooperative Oncology Group) and KPS (Karnofsky Performance Status).^18^, ^19^

### Statistical analysis

The values obtained for each continuous variable were described by the mean and Standard Deviation (SD) or by the median followed by the amplitude, the interquartile distance (difference between the 25th and 75th percentiles) or by the 95% Confidence Interval (95% CI). Relative and absolute frequencies were used to describe qualitative data. The distributions were defined as parametric or nonparametric by the Kolmogorov–Smirnov test. Comparisons of the frequency of a phenomenon between groups of qualitative variables were performed using Fisher's exact test or the Chi-Square test. In the comparisons of a quantitative variable between two nonparametric sample populations, the Mann–Whitney test was used, and Student’s *t*-test was used in the parametric populations. All analyses were performed with SPSS version 24.0 (SPSS® Inc., Illinois, USA) and *p*-values less than 5% (*p* < 0.05) were considered significant.

## Results

The evaluation of quality of life with the application of the University of Washington questionnaire was completed by 24 patients: 15 received SLB and 9 had SND. The characteristics of both groups are shown in Table 1. It was observed that patients who received SND had relatively larger (*p* < 0.001) and thicker (*p* = 0.001) tumors, greater pathological stages (*p* = 0.001), more neck metastases (*p* = 0.003) and received more adjuvant radiotherapy (*p* = 0.004).

**Table 1:** Descriptive data and analysis of the homogeneity of the quality of life evaluation from the two groups of patients that received either a Selective Neck Dissection (SND) or a Sentinel Lymph node Biopsy (SLB).

Variable	SLB	SND	*p*
Demographic data			
Male	10 (66.7%)	9 (100.0%)	0.118^b^
Age (years)^a^	62.6 ± 10.0	62.2 ± 9.3	0.953
Subsite of primary tumor	–	–	
Retromolar trigone	–	4 (44.4%)	0.002^c^
Lip	–	1 (11.1%)	
Tongue	13 (86.7%)	1 (11.1%)	
Hard Palate	1 (6.7%)	–	
Floor of Mouth	1 (6.7%)	3 (33.3%)	
Clinical T-stage			
T1	12 (80.0%)	–	<0.001^b^
T2	3 (20.0%)	9 (100.0%)	
Tumor characteristics			
Perineural invasion	1 (7.1%)	2 (22.2%)	0.538^b^
Angiolymphatic invasion			
Tumor thickness (CM)	0.6 ± 0.4	1.0 ± 0.5	0.001
Pathological T-stage	–	–	–
pT1	14 (93.3%)	2 (22.2%)	0.001^c^
pT2	1 (6.7%)	5 (55.6%)	
pT3	–	1 (11.1%)	
pT4a	–	1 (11.1%)	0.003^b^
Lymph node metastases	–	5 (55.6%)	–
Extracapsular spread	–	2 (40.0%)	
Final Stage			<0.001^c^
I	14 (93.3%)	1 (11.1%)	
II	1 (6.7%)	3 (33.3%)	
IV	–	5 (55.6%)	
Follow-up			
Adjuvant radiotherapy	1 (6.7%)	6 (66.7%)	0.004^b^
Concurrent chemotherapy	–	1 (11.1%)	0.375^b^
Follow-up period (months)	31.7 ± 7.8	38.0 ± 5.9	–

a Mean ± standard deviation.

b Fisher’s exact test.

c Chi-Square test and Mann–Whitney test in the other analyzes.

The comparison of the performance status scores evaluated by the ECOG and KPS scales, although not statistically significant, showed a greater delayed commitment of patients who underwent SND when compared to those receiving SLB (for SLB = 93.3% were classified as ECOG 0 and 6.7% as ECOG 1 *vs.*, respectively, 66.7% and 33.3% for SND).

The evaluation of the UW-QOL questionnaire showed a worsening of the domains of appearance (*p* = 0.035) and of chewing (*p* = 0.041), as well as a decrease of approximately 10% in the final mean (*p* = 0.025) of patients who received SND when compared to those receiving SLB. Complete data from all domains on the quality of life questionnaire are detailed in Table 2.

**Table 2:** Comparative evaluation of the domains of the UW-QOL questionnaire in the groups receiving either a Selective Neck Dissection (SND) or a Sentinel Lymph node Biopsy (SLB).

Domain	SLB	SND	*p*^a^
Pain	88.3 ± 22.9	91.7 ± 17.7	0.861
Appearance	93.3 ± 14.8	80.6 ± 11.0	0.035
Activity	86.7 ± 29.7	83.3 ± 17.7	0.318
Recreation	90.0 ± 26.4	86.1 ± 18.2	0.379
Deglutition	93.3 ± 18.7	89.0 ± 16.5	0.482
Chewing	86.7 ± 22.9	56.7 ± 30.0	0.041
Speech	86.8 ± 16.7	81.7 ± 17.4	0.558
Shoulder	91.1 ± 26.6	85.3 ± 17.4	0.263
Palate	82.2 ± 33.1	59.2 ± 36.5	0.138
Saliva	84.5 ± 30.5	63.0 ± 26.2	0.055
Humor	91.7 ± 18.1	88.9 ± 18.2	0.682
Anxiety	91.2 ± 15.1	81.6 ± 24.2	0.411
Final mean	88.8 ± 18.0	78.9 ± 11.5	0.025

Values described by the mean ± standard deviation.

a Mann–Whitney test.

In the portion of the qualitative evaluation from the UW-QOL questionnaire about the last seven days, most patients in both groups classified their quality of life as “good” (53.3% for SLB and 66.7% for SND), but none of the patients receiving SND reported an “excellent” quality of life (26.7% for SLB) and only one (11.1%) was reported as “very good” (13.3% for SLB). On the other hand, as demonstrated, forty percent of the patients receiving SLB reported their current quality of life as “excellent” or “very good”.

Patients were questioned about everything that contributes to the individual's quality of life over the last seven days, which includes not only physical and mental health, spirituality, and personal leisure but also many other factors, such as family, friends and activities that are important to satisfaction with life. The patient’s responses were similar to those in the previous question; none of the patients receiving SND reported an “excellent” or “very good” quality of life, unlike patients receiving SLB, where 46.6% of patients referred to their current general quality of life as “excellent” or “very good” (respectively, 33.3% and 13.3% for SLB *vs.* 66.7% and 33.3% for SND).

## Discussion

The present study evaluated late quality of life in patients receiving SND or SLB for oral cavity SCC. Patients undergoing SLB demonstrated a superiority of appearance and chewing and an approximate 10% increase in their quality of life mean score as measured by the UW-QOL questionnaire. Moreover, some other qualitative indexes also demonstrated that the SLB was superior to the SND in terms of late quality of life evaluation.

Neck dissection has had a positive impact on the survival of patients with head and neck cancer for almost a century.^20^ The procedure was so iconic that it became a symbol of head and neck surgery.^21^ However, since its initial description in English literature in 1906 by George W. Crile,^22^ proposals for modifications derived from the original surgery, classical radical neck dissection, have arisen. These changes were aimed at limiting the extent of resection of both lymphatic and nonlymphatic structures, aiming to reduce the mortality and morbidity associated with the procedure.^23^, ^24^

In the case of patients with initial tumors of the oral cavity and clinically negative neck lymph nodes, occult metastases may occur in 20%–30% of the cases and the number of patients who would not benefit from SND corresponds to more than two-thirds of the cases. Moreover, some of them will also suffer complications from the procedure.^25^ The SLB has the requirements to fill this gap, since its purpose is to promote the surgical removal of lymph nodes truly at risk for disease, sparing the patient from wider, unnecessary neck dissection and minimizing the risk of treatment-associated sequelae.^11^, ^26^

Treatment for head and neck cancer, because of its anatomical location, can lead to significant changes in vital functions related to feeding, communication, and the social interaction of individuals. Considering this scenario, the treatments proposed in the last decades also aim to minimize morbidity for each patient. This morbidity may or may not result in an altered quality of life. In this sense, quality of life questionnaires are of fundamental importance, incorporating the patient's point of view and focusing the treatment on the patient rather than on the disease.^8^, ^27^

In this study, all patients were evaluated at least 36 months after the end of cancer treatment. During this period, the sequelae were minimized by the adaptation itself and by rehabilitation programs helping to stabilize the answers in the questionnaire.^28^

The two groups presented similar characteristics in relation to age and gender, factors that frequently influence the responses of the questionnaire. Males and young individuals are known to have better scores on quality of life tests.^29^

There was a significant difference between groups regarding quality of life. Statistically, the SLB group had a higher quality of life than the SND group. Additionally, in the performance status tests of KPS and ECOG, as well as in the qualitative questions of the questionnaire, although not statistically significant, we could see a trend towards the difference between the groups corroborating the findings of a significant better quality of life in patients receiving SLB.

This difference in worse quality of life related to the neck dissection group, could be related to more radiation therapy in this group, as more aggressive disease was present in the neck dissection group. There was no significant difference in relation to the domains questioned between the two groups, except in appearance and chewing. The SND group presented a worse quality of life in both domains. The findings regarding appearance may be explained by the cervical incision itself, a major aesthetic alteration. Regarding chewing, patients who receiving SND also had significantly lower scores on the questionnaire. This may be associated with the fact that the SND group presented a greater need for postoperative adjuvant treatment. According to the literature, radiotherapy is the major cause of this symptom. However, radiotherapy does not only compromise chewing. In the saliva and swallowing domains, differences between groups would also be expected.^30^

In addition to the difference observed for adjuvant treatment, there was also a disproportion between the groups in relation to the pathological staging being able to contribute to the results found. In the SLB group, pT1 cases predominated, unlike the SND group, where pT2 cases predominated. This difference is explained by the fact that many T2 cases were allocated into the SND group because, due to the thickness of the primary tumor, an oncologically safe transoral resection would not be possible, thus requiring a combined oral and cervical approach, which is an exclusion condition for the performance of SLB in our hospital. Unfortunately, due the reduced number of enrolled patients in the present study, once patients in early stage of oral cancer are very few, it was not possible to perform a multivariate analysis or a prospective trial in order to better explain the impact of different clinical, pathological and demographic data as confounding factors in the QOL of patients submitted to SLB or SND.

It is important to note that the domain related to the patient's shoulder showed no significant difference between the groups. Accessory nerve injury is one of the risks of SND that is minimized in SLB. However, these findings can be attributed to both the patient's perception of what represents “shoulder pain” and to the small number of patients evaluated. This reduced number reflects a strict percentage of patients with squamous cell carcinoma of the oral cavity that are included in the study, since the presentation in the early stages represents a minority of the cases diagnosed in our country.

## Conclusion

In conclusion, patients with early oral cavity squamous cell carcinoma that received a sentinel lymph node biopsy presented better late results for general quality of life, mainly regarding appearance and chewing, when compared to patients receiving a selective neck dissection.

## Conflicts of interest

The authors declare no conflicts of interest.
